# Sjögren’s Syndrome-Associated Medium- and Large-Vessel Vasculitis Presenting With Acute Abdominal Symptoms

**DOI:** 10.7759/cureus.116446

**Published:** 2026-09-17

**Authors:** Mohammed Mahgoub, Hala Najjari, Abdelmoneim Musa

**Affiliations:** 1 Internal Medicine, Shaikh Khalifa Medical City, Ajman, ARE; 2 Medical Education, Ajman University, Ajman, ARE

**Keywords:** acute abdominal symptoms, large-vessels vasculitis, medium-vessels vasculitis, sjogren syndrome, vasculitis

## Abstract

We report a case of Sjögren’s syndrome-associated vasculitis involving the medium and large arteries in a patient who presented with acute abdominal pain, vomiting, and fever. Computed tomography angiography demonstrated vasculitic involvement of the superior mesenteric, inferior mesenteric, renal, and left external iliac arteries. Immunological evaluation revealed strongly positive antinuclear antibodies (ANA) and an extractable nuclear antigen (ENA) profile positive for anti-SSA, anti-SSB, and Ro-52 antibodies, while anti-double-stranded DNA (anti-dsDNA) antibodies were negative. The patient responded rapidly to systemic corticosteroid therapy and was discharged on oral treatment. This case highlights a rare presentation of Sjögren’s syndrome with medium- and large-vessel vasculitis manifesting as acute abdominal symptoms.

## Introduction

Sjögren’s syndrome is a chronic systemic autoimmune disease characterized by lymphocytic infiltration of the exocrine glands, resulting in xerostomia and keratoconjunctivitis sicca [1]. Although glandular involvement is the hallmark of the disease, extraglandular manifestations occur in a substantial proportion of patients and may involve the musculoskeletal, pulmonary, renal, neurological, and vascular systems [2]. Vasculitis is a well-recognized systemic manifestation and most commonly affects small vessels [3]. Medium- and large-vessel involvement is uncommon but may result in severe, life-threatening complications, including mesenteric ischemia, renal ischemia, and other vascular emergencies [4]. Recognition of these atypical manifestations is essential to facilitate timely diagnosis and treatment.

## Case presentation

A 39-year-old Ethiopian woman with a three-year history of hypertension, treated with atenolol and amlodipine, presented to the emergency department in April 2024 with acute-onset colicky mid-abdominal and right flank pain associated with vomiting, fever, and fatigue. She reported recurrent episodes of abdominal pain since May 2022 without an established diagnosis. There was no history of nephrolithiasis, Raynaud's phenomenon, oral ulcers, skin rash, or previous thromboembolic disease. On further questioning, she described a longstanding history of dry mouth but denied significant dry eyes.

On presentation, the patient appeared uncomfortable because of abdominal pain but was alert and oriented. She was hemodynamically stable and febrile. Abdominal examination revealed mild tenderness over the periumbilical and right flank regions without guarding, rebound tenderness, or abdominal distension. Bowel sounds were present and normal. There was no palpable abdominal mass or organomegaly. Cardiovascular and respiratory examinations were otherwise unremarkable, with no audible bruits or signs of heart failure. Peripheral pulses were palpable and symmetrical, and there were no skin rashes, purpura, digital ischemia, or peripheral edema. Neurological examination was normal.

Initial laboratory workup was unremarkable apart from slightly raised serum creatinine and raised ESR, as shown in Table 1.

**Table 1:** Laboratory investigations at initial presentation

Investigation	Result	Reference Range	Interpretation
Haemoglobin	11.6 g/dL	12.0–15.5 g/dL (female)	Mild anaemia
White blood cell count	7.76 × 10⁹/L	4.0–11.0 × 10⁹/L	Within normal limits
Platelet count	232 × 10⁹/L	150–450 × 10⁹/L	Within normal limits
Erythrocyte sedimentation rate (ESR)	42 mm/h	<20 mm/h (female)	Elevated
Serum creatinine	95 µmol/L	45–90 µmol/L (female)	Borderline elevated/near normal
Sodium	133 mmol/L	135–145 mmol/L	Mild hyponatraemia
Potassium	4.1 mmol/L	3.5–5.1 mmol/L	Within normal limits
Lactic acid	1.0 mmol/L	0.5–2.2 mmol/L	Normal
Amylase	82 U/L	28–100 U/L	Normal
Hepatitis B surface antigen	Non-reactive	Non-reactive	Negative
Hepatitis C antibody	Non-reactive	Non-reactive	Negative
Treponema pallidum serology	Non-reactive	Non-reactive	Negative

Contrast-enhanced computed tomography (CT) of the abdomen demonstrated focal perivascular fat stranding surrounding the root of the superior mesenteric artery (SMA) with associated focal arterial wall thickening, raising suspicion for SMA vasculitis. No significant luminal stenosis or occlusion was identified. The examination also demonstrated a simple right ovarian cyst, while the kidneys appeared normal without evidence of renal masses, calculi, hydronephrosis, or obstructive uropathy.

The patient was evaluated by the surgical team, who found no clinical or radiological evidence of acute bowel ischemia or other indications for surgical intervention. She initially left the hospital against medical advice but returned the following day because of persistent symptoms. She was admitted for supportive management with intravenous fluids, analgesics, antiemetics, empirical intravenous antibiotics, and oral prednisolone (1 mg/kg/day).

CT angiography of the abdomen performed in April 2024 demonstrated inflammatory changes involving multiple vessels, including persistent mural thickening and surrounding inflammatory fat stranding of the proximal SMA (Figure 1), focal wall thickening of the inferior mesenteric artery (IMA), irregularity of the abdominal aortic wall, bilateral moderate-to-severe ostial renal artery stenosis with bilateral renal cortical scarring (Figure 2), and focal circumferential thickening of the left external iliac artery. These findings were considered highly suggestive of medium- and large-vessel vasculitis.

**Figure 1:**
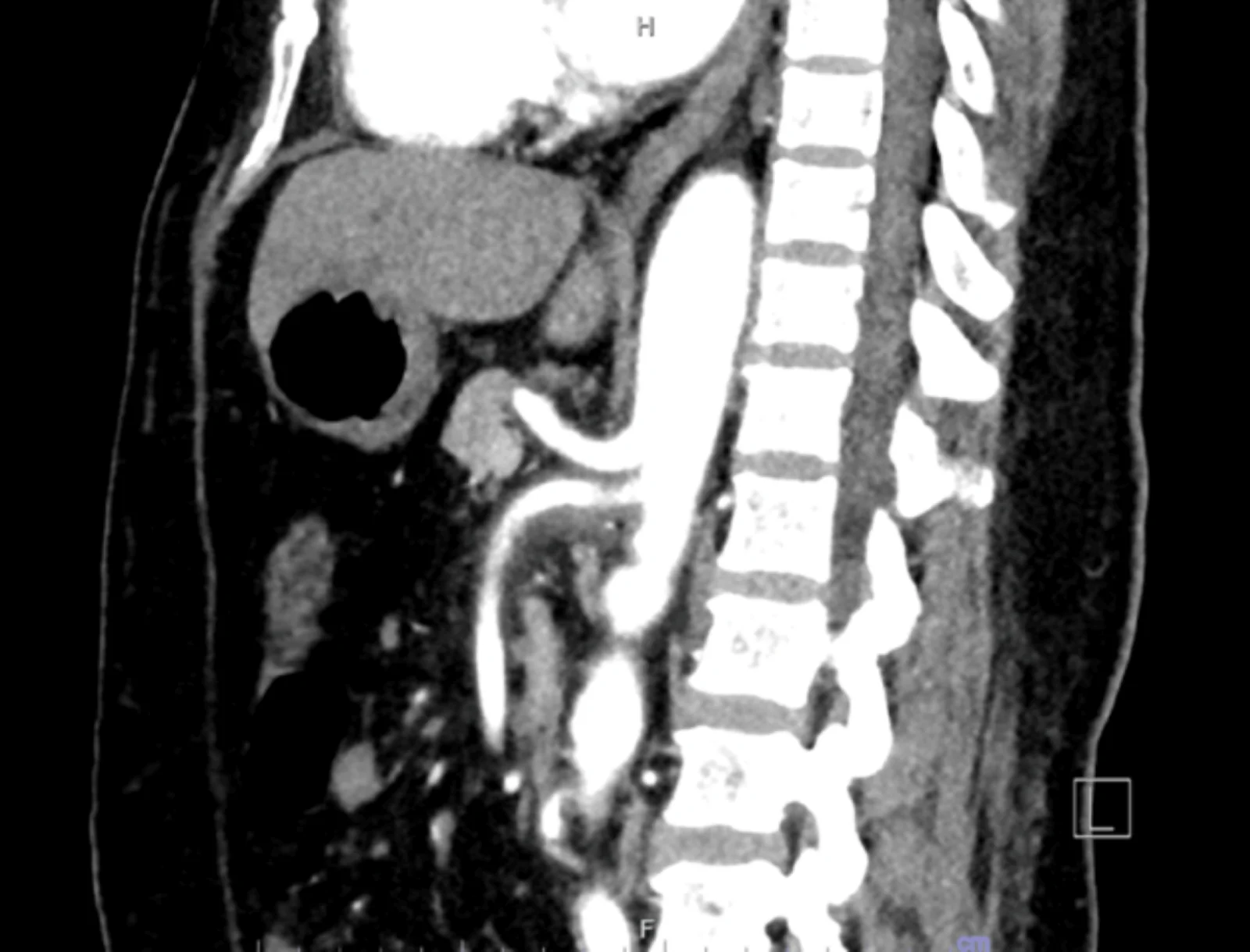
CT angiography abdomen in sagittal view shows the superior mesenteric artery (SMA) origin stenosis

**Figure 2:**
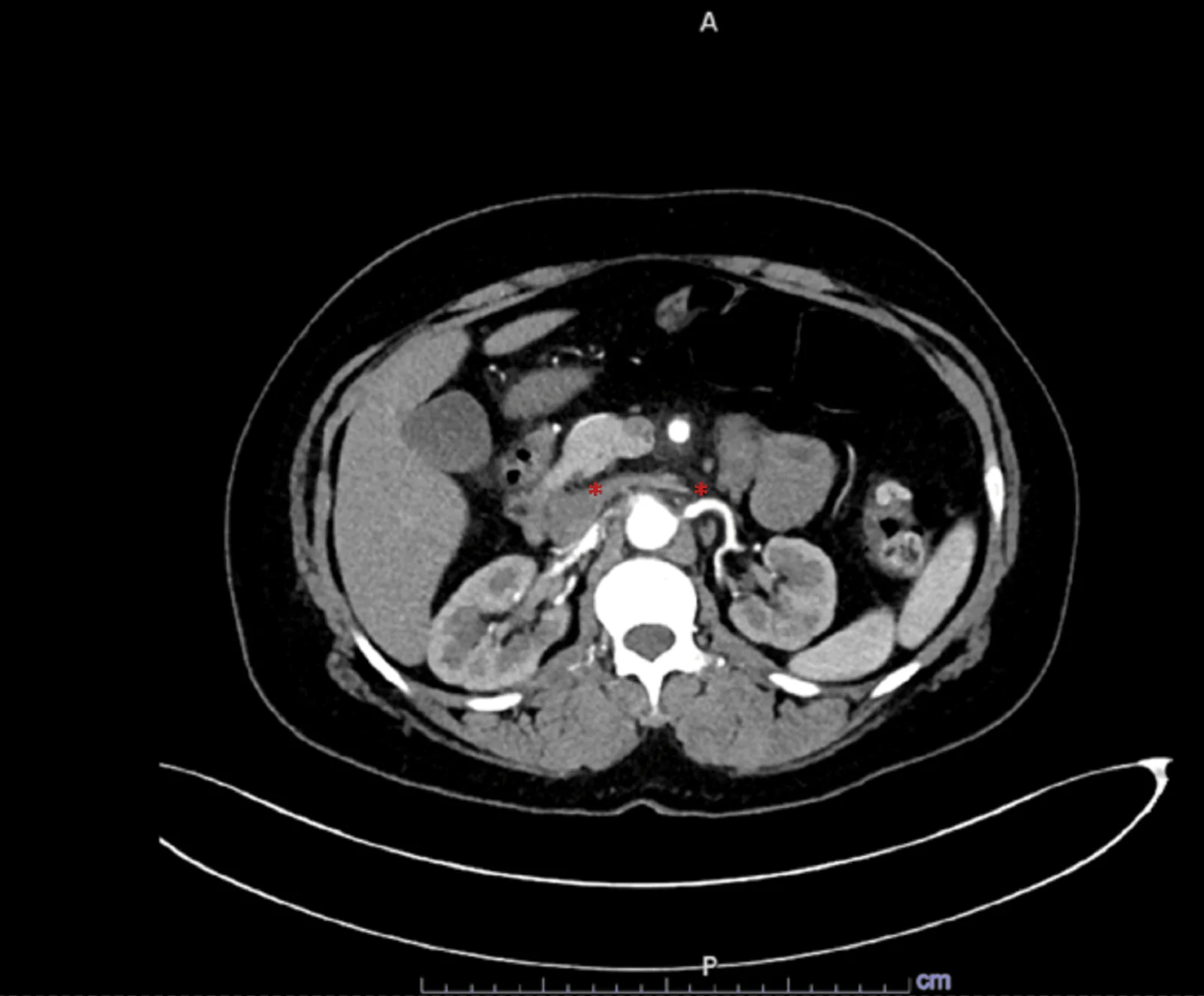
CT angiography of the abdomen demonstrating bilateral renal scarring with bilateral moderate-to-severe ostial renal artery stenosis (red asterisks)

The patient remained hemodynamically stable without evidence of bowel infarction or organ ischemia. Her abdominal pain improved following corticosteroid therapy, and she was discharged in the same month, April 2024, with oral prednisolone, outpatient autoimmune investigations, and rheumatology follow-up.

At a review in the internal medicine clinic in May 2024, she reported marked symptomatic improvement. Physical examination demonstrated xerostomia with bilateral, non-tender parotid gland enlargement supporting the diagnosis of primary Sjögren's syndrome. Ocular examination revealed no clinically apparent conjunctival injection or evidence of significant keratoconjunctivitis sicca. There were no oral ulcers, synovitis, Raynaud's phenomenon, or cutaneous manifestations of systemic vasculitis. Autoimmune investigations revealed a strongly positive extractable nuclear antigen (ENA) screen (>429.4; reference <20), positive antinuclear antibodies (ANA), and positive anti-SSA/Ro, anti-SSB/La, and Ro-52 antibodies, while anti-double-stranded DNA antibodies were negative (Table 2).

**Table 2:** Autoimmune serological investigations

Investigation	Result	Reference Range	Interpretation
Antinuclear antibody (ANA)	Positive	Negative	Suggestive of autoimmune connective tissue disease
Extractable nuclear antigen (ENA) titre	>429.4	<20	Markedly elevated
Anti-SSA (Ro) antibody	Positive	Negative	Strongly associated with primary Sjögren's syndrome and connective tissue disease
Anti-SSB (La) antibody	Positive	Negative	Associated with primary Sjögren's syndrome
Ro-52 antibody	Positive	Negative	Associated with primary Sjögren's syndrome and other connective tissue diseases
Anti-double-stranded DNA (anti-dsDNA) antibody	Negative	Negative	Less supportive of systemic lupus erythematosus
Anti-nucleosome antibody	Negative	Negative	Negative
Anti-histone antibody	Negative	Negative	Negative
Anti-ribonucleoprotein (anti-RNP) antibody	Negative	Negative	Negative
Rheumatoid factor (IgM)	Negative	Negative	Negative

Approximately four months later, in September 2024, she presented with worsening exertional chest pain and dyspnea of six months' duration, which had acutely deteriorated during the preceding week. She admitted poor adherence to corticosteroid therapy and had missed scheduled rheumatology follow-up appointments. Electrocardiography demonstrated lateral T-wave inversion, while laboratory investigations revealed markedly elevated cardiac troponin (7,398 ng/L; reference 0-34 ng/L) with mildly elevated CK-MB (0.1 ng/mL; reference 0-0.06 ng/mL). Transthoracic echocardiography demonstrated lateral wall hypokinesia with a left ventricular ejection fraction of 45%. She was diagnosed with non-ST-elevation myocardial infarction (NSTEMI) (GRACE score 80) and referred for coronary angiography.

During the procedure, vascular access proved difficult because of diminished peripheral pulses. Angiography demonstrated complete occlusion of the proximal left subclavian artery, and the coronary angiogram was abandoned because guidewire advancement was unsuccessful. Given these unexpected findings, coronary CT angiography with three-dimensional reconstruction was performed instead.

Coronary CT angiography demonstrated normal coronary arteries without evidence of atherosclerosis or stenosis. However, extensive medium- and large-vessel vasculitis was identified involving the thoracoabdominal aorta, left subclavian artery, bilateral renal arteries, SMA, IMA, bilateral internal iliac arteries, and bilateral superficial femoral arteries (Figure 3). Additional findings included complete occlusion of the proximal left subclavian artery, bilateral renal artery stenoses and occlusions, 70% stenosis of the proximal IMA, bilateral internal iliac artery occlusions, segmental occlusion of the right superficial femoral artery, diffuse stenotic disease of the left superficial femoral artery, and mild dilatation with mural thickening of the SMA. A small calcified granuloma was also noted in the left lower lobe of the lung.

**Figure 3:**
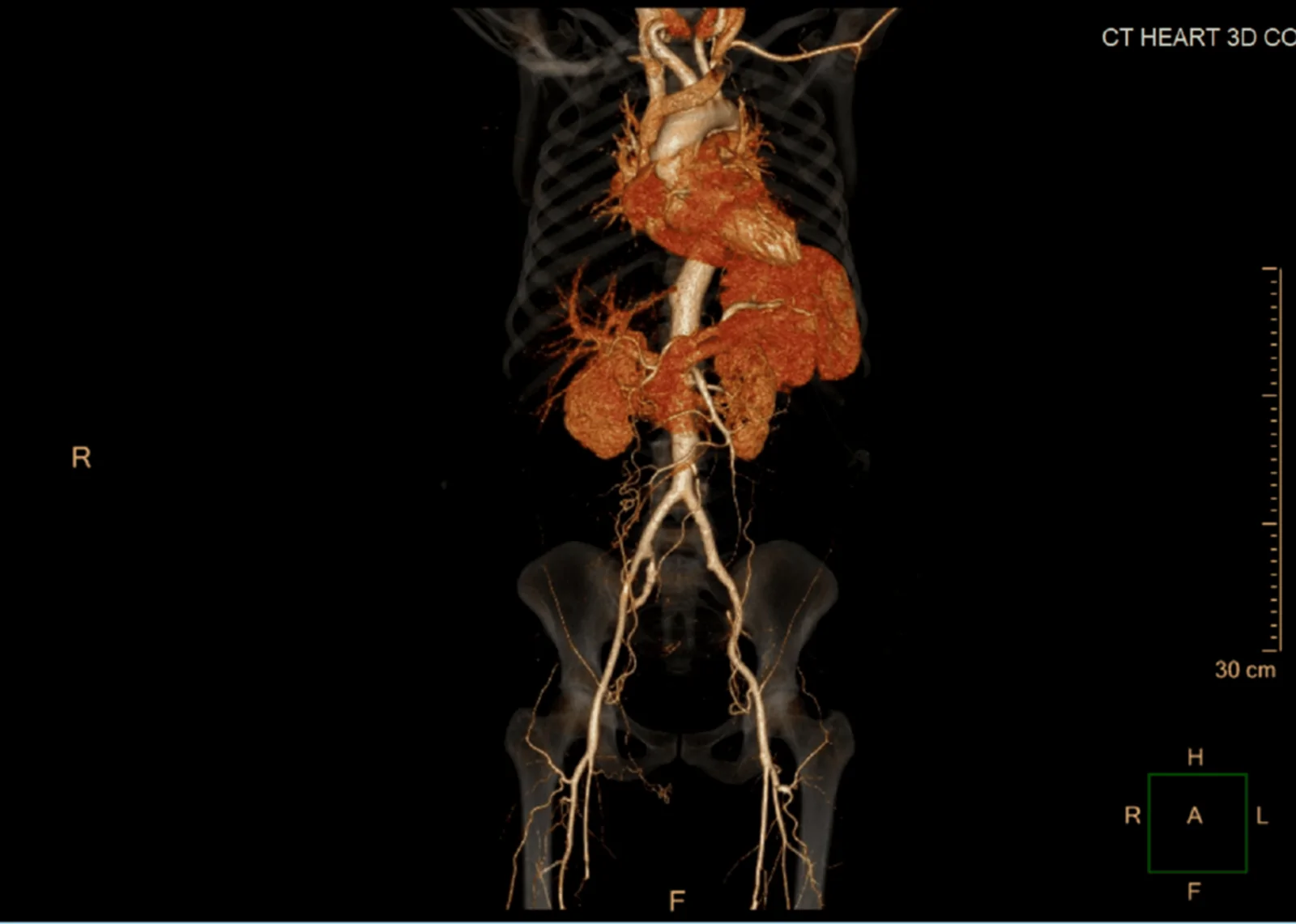
Three-dimensional CT angiography Three-dimensional CT angiographic reconstruction demonstrating widespread medium- and large-vessel vasculitis. The image demonstrates luminal irregularity of the thoracoabdominal aorta, proximal occlusion of the left subclavian artery, severe bilateral renal artery ostial stenoses, mural thickening of the superior mesenteric artery, and involvement of the iliac and femoral arteries, consistent with extensive systemic vasculitis.

**Table 3:** Summary of radiological investigations SMA: superior mesenteric artery; IMA: inferior mesenteric artery

Date	Imaging modality	Key findings	Clinical significance
April 2024	Contrast-enhanced CT abdomen	Perivascular fat stranding surrounding the root of the SMA with focal mural thickening; no significant luminal stenosis or occlusion. Incidental simple right ovarian cyst. No bowel obstruction, appendicitis, renal calculi, hydronephrosis, or abdominal lymphadenopathy.	Initial suspicion of isolated SMA vasculitis without bowel ischemia.
April 2024 (three days after initial presentation)	CT angiography of the abdomen	Persistent inflammatory thickening of the SMA with surrounding fat stranding; focal wall thickening of the IMA; irregular abdominal aortic wall; bilateral moderate-to-severe ostial renal artery stenosis with bilateral renal cortical scarring; focal circumferential thickening of the left external iliac artery; calcified left lower lobe pulmonary granuloma.	Demonstrated progression from isolated mesenteric involvement to multisite medium- and large-vessel vasculitis.
September 2024	Coronary CT angiography with 3D reconstruction	Normal coronary arteries without atherosclerosis or stenosis. Large- and medium-vessel vasculopathy involving the thoracoabdominal aorta, left subclavian artery, bilateral renal arteries, SMA, IMA, bilateral internal iliac arteries, and bilateral superficial femoral arteries. Complete occlusion of the left subclavian artery; bilateral renal artery stenoses/occlusions; 70% proximal IMA stenosis; bilateral internal iliac occlusions; right superficial femoral artery occlusion; diffuse left superficial femoral artery narrowing. Left ventricular hypertrophy and a calcified granuloma in the left lower lobe.	Confirmed extensive systemic medium- and large-vessel vasculitis with disease progression despite normal coronary arteries.

The extensive progression of arterial involvement despite normal coronary arteries supported the diagnosis of systemic medium- and large-vessel vasculitis associated with primary Sjögren's syndrome. She was discharged in September 2024 in stable condition. Anti-ischemic medications were prescribed, and she was advised to follow up with rheumatology.

## Discussion

Primary Sjögren's syndrome is a chronic autoimmune disease characterized by lymphocytic infiltration of the exocrine glands, resulting in xerostomia and keratoconjunctivitis sicca. Approximately one-third of patients develop extraglandular manifestations involving the musculoskeletal, pulmonary, renal, neurological, or vascular systems [1,2].

Our patient demonstrated an unusual pattern of widespread medium- and large-vessel vasculitis involving the superior mesenteric artery, inferior mesenteric artery, bilateral renal arteries, left external iliac artery, bilateral internal iliac arteries, bilateral superficial femoral arteries, thoracoabdominal aorta, and left subclavian artery. The diagnosis was supported by CT angiographic findings together with strongly positive ANA, anti-SSA/Ro, anti-SSB/La, and Ro-52 antibodies. Although the patient reported only longstanding dry mouth without significant ocular symptoms at initial presentation, subsequent examination demonstrated bilateral parotid enlargement, supporting the diagnosis of Sjögren's syndrome. This presentation illustrates that severe systemic vasculitis may precede or overshadow the classic sicca manifestations of the disease.

Vasculitis is among the most serious systemic complications and most commonly affects small vessels, manifesting as palpable purpura, cutaneous leukocytoclastic vasculitis, peripheral neuropathy, or cryoglobulinemic disease. Medium-vessel vasculitis accounts for fewer than 5% of reported vasculitic manifestations, whereas large-vessel involvement remains exceptionally rare and is largely confined to isolated case reports and small case series [4,5]. Small-vessel vasculitis is believed to result predominantly from immune-complex deposition and B-cell hyperactivity associated with hypergammaglobulinemia, rheumatoid factor positivity, hypocomplementemia, and cryoglobulinemia [3].

The pathogenesis of large-vessel vasculitis in Sjögren's syndrome remains incompletely understood. Some authors have proposed that these patients may represent an overlap autoimmune phenotype sharing pathogenic mechanisms with primary large-vessel vasculitides such as Takayasu arteritis [6]. Medium- and large-vessel vasculitis is a rare but important manifestation that should be considered in patients presenting with unexplained abdominal pain and positive autoimmune serology [7,8]. Large-vessel disease may involve additional mechanisms, including chronic endothelial immune activation, B-cell-mediated vascular inflammation, type I interferon pathway activation, and inflammatory remodeling of the arterial wall [9,10]. 

Diagnosis is frequently delayed because constitutional symptoms and inflammatory markers are nonspecific, while classic sicca symptoms may be absent or mild at presentation [2,9]. Cross-sectional vascular imaging plays a central role in identifying arterial wall thickening, mural enhancement, stenosis, occlusion, aneurysm formation, and inflammatory changes while excluding alternative causes such as atherosclerosis or embolic disease [6-8]. In our patient, serial CT angiography not only established the diagnosis but also demonstrated progression of vascular disease involving additional arterial territories.

Several published reports have described large-vessel vasculitis associated with Sjögren's syndrome, although the anatomical distribution has varied considerably. Zhu et al. reported a series of patients presenting with ischemic stroke or transient ischemic attack secondary to cervical and intracranial large-vessel vasculitis, highlighting that cerebrovascular disease may be the initial manifestation of primary Sjögren's syndrome [11]. Similarly, Saied et al. described cerebral large-vessel vasculitis occurring after Guillain-Barré syndrome as the first presentation of primary Sjögren's syndrome [12]. Unlike these reports, our patient had no neurological manifestations but instead presented with acute abdominal pain due to mesenteric arterial inflammation.

Mesenteric vascular involvement has been reported only rarely in Sjögren's syndrome. Rios-Fernández et al. described mesenteric inflammatory veno-occlusive disease resulting in intestinal ischemia in a patient with Sjögren's syndrome [13]. In contrast, our patient had arterial rather than venous involvement affecting both the superior and inferior mesenteric arteries, without radiological evidence of bowel infarction. Early recognition and prompt corticosteroid treatment likely prevented progression to irreversible mesenteric ischemia.

There are currently no randomized controlled trials guiding treatment of Sjögren's syndrome-associated large-vessel vasculitis. Management is extrapolated from severe systemic vasculitis and extraglandular Sjögren's syndrome, with high-dose glucocorticoids forming the cornerstone of induction therapy [6]. Rituximab, cyclophosphamide, azathioprine, or mycophenolate mofetil may be required in refractory or extensive disease [2,4,11]. Our patient demonstrated rapid improvement in abdominal symptoms following initiation of systemic corticosteroids, emphasizing the importance of early immunosuppressive therapy. However, subsequent progression of vascular disease coincided with poor treatment adherence and loss to rheumatology follow-up, highlighting the importance of long-term monitoring and maintenance immunosuppression in preventing irreversible vascular injury.

Another distinguishing feature of our case is the progressive multisite arterial involvement documented on serial imaging. Initial CT angiography demonstrated inflammatory changes involving the superior mesenteric artery, inferior mesenteric artery, renal arteries, aorta, and external iliac artery. Follow-up cardiovascular CT several months later demonstrated progression with complete occlusion of the left subclavian artery, bilateral renal artery stenosis and occlusion, bilateral internal iliac artery occlusion, superficial femoral artery disease, and persistent mesenteric arterial involvement despite normal coronary arteries. Such extensive vascular involvement across multiple arterial territories has only rarely been reported in patients with Sjögren's syndrome [14].

The differential diagnosis included Takayasu arteritis, polyarteritis nodosa, ANCA-associated vasculitis, giant cell arteritis, infectious aortitis, and IgG4-related disease. Takayasu arteritis was considered because of the patient's age and involvement of the aorta and its major branches. However, the presence of strongly positive anti-SSA and anti-SSB antibodies, longstanding xerostomia, bilateral parotid enlargement, and the absence of clinical features typically associated with Takayasu arteritis favored Sjögren's syndrome-associated vasculitis. Polyarteritis nodosa was considered less likely because there was no evidence of microaneurysms, hepatitis B infection, or characteristic multisystem involvement. Negative anti-double-stranded DNA antibodies also made systemic lupus erythematosus less likely. Although ANCA testing, complement levels, and cryoglobulin analysis would have further strengthened exclusion of alternative vasculitic disorders, the combination of clinical, serological, and radiological findings strongly supported Sjögren's syndrome-associated systemic vasculitis [15].

Compared with previously reported cases (Table 4), our patient is unique because of the combination of acute abdominal presentation, extensive involvement of both medium- and large-sized arteries, progression to subclavian and peripheral arterial occlusive disease documented on serial imaging, preserved coronary arteries despite presentation with non-ST elevation myocardial infarction, and strongly positive Sjögren-specific autoantibodies despite only subtle sicca symptoms. This broad spectrum of vascular involvement expands the recognized phenotype of Sjögren's syndrome-associated vasculitis and underscores the importance of considering autoimmune disease in patients presenting with otherwise unexplained multisite arterial inflammation.

**Table 4:** Comparison of published cases of Sjögren syndrome-associated large-vessel vasculitis SS: Sjögren syndrome; TIA: transient ischemic attack; NSTEMI: non-ST-elevation myocardial infarction; SMA: superior mesenteric artery; IMA: inferior mesenteric artery; SSA/SSB: Sjögren syndrome-related antigen A/Sjögren syndrome-related antigen B

Study	Initial presentation	Vessels involved	Sjögren diagnosis	Treatment	Outcome
Zhu et al., 2024 [11]	Stroke/TIA	Cervical and intracranial arteries	Primary SS	Steroids + immunosuppressants	Neurological improvement
Saied et al., 2024 [12]	Guillain-Barré syndrome followed by stroke	Cerebral large vessels	Primary SS	Steroids + immunosuppression	Clinical improvement
Rios-Fernández et al., 2014 [13]	Intestinal ischemia	Mesenteric veins	Primary SS	Surgery + steroids	Recovery
Present case	Acute abdominal pain followed by NSTEMI	SMA, IMA, aorta, bilateral renal, subclavian, iliac and femoral arteries	Primary SS with positive SSA/SSB	Corticosteroids	Initial improvement; later progression associated with poor adherence

Learning points

Sjögren’s syndrome may initially present with systemic vasculitis despite minimal or absent classic sicca symptoms [2]. Although rare, medium- and large-vessel vasculitis is an important manifestation that should be considered in patients presenting with unexplained abdominal pain and positive autoimmune serology [7-8]. CT angiography is an essential diagnostic modality for identifying vascular involvement and guiding appropriate management [6-8]. Early initiation of immunosuppressive therapy can lead to rapid clinical improvement and may help prevent irreversible vascular and end-organ complications [8].

## Conclusions

This case highlights the rare occurrence of Sjögren’s syndrome-associated vasculitis involving the medium and large arteries, presenting as acute abdominal pain, vomiting, and fever. The atypical presentation and extensive vascular involvement underscore the importance of maintaining a high index of suspicion for systemic vasculitis in patients with Sjögren’s syndrome who present with unexplained acute abdominal symptoms. Prompt recognition through appropriate imaging and serological evaluation, followed by timely initiation of immunosuppressive therapy, is essential to prevent potentially life-threatening vascular complications and improve clinical outcomes. This case expands the limited literature on large-vessel involvement in Sjögren’s syndrome and emphasizes the need for multidisciplinary evaluation and individualized management in patients with atypical vasculitic manifestations.
